# Plasticity in the Visual System is Associated with Prosthesis Use in Phantom Limb Pain

**DOI:** 10.3389/fnhum.2013.00311

**Published:** 2013-06-24

**Authors:** Sandra Preißler, Caroline Dietrich, Kathrin R. Blume, Gunther O. Hofmann, Wolfgang H. R. Miltner, Thomas Weiss

**Affiliations:** ^1^Department of Biological and Clinical Psychology, Institute of Psychology, Friedrich Schiller University, Jena, Germany; ^2^Berufsgenossenschaftliche Kliniken Bergmannstrost Halle/Saale, Halle, Germany; ^3^Department of Trauma, Hand and Reconstructive Surgery, University Hospital Jena, Jena, Germany

**Keywords:** phantom limb pain, magnetic resonance imaging, morphometry, use of myoelectric prostheses, visual stream

## Abstract

The experience of strong phantom limb pain (PLP) in arm amputees was previously shown to be associated with structural neural plasticity in parts of the cortex that belong to dorsal and ventral visual streams. It has been speculated that this plasticity results from the extensive use of a functional prosthesis which is associated with increased visual feedback to control the artificial hand. To test this hypothesis, we reanalyzed data of cortical volumes of 21 upper limb amputees and tested the association between the amount of use of the hand prosthesis and cortical volume plasticity. On the behavioral level, we found no relation between PLP and the amount of prosthesis use for the whole patient group. However, by subdividing the patient group into patients with strong PLP and those with low to medium PLP, stronger pain was significantly associated with less prosthesis use whereas the group with low PLP did not show such an association. Most plasticity of cortical volume was identified within the dorsal stream. The more the patients that suffered from strong PLP used their prosthesis, the smaller was the volume of their posterior parietal cortex. Our data indicate a relationship between prosthesis use and cortical plasticity of the visual stream. This plasticity might present a brain adaptation process to new movement and coordination patterns needed to guide an artificial hand.

## Introduction

After amputation and deafferentation of a limb, up to 98% of individuals report vivid sensations in the missing body part (Ramachandran and Hirstein, 1998). In turn, up to 80% of amputees describe these sensations as painful (Flor, 2002; Ephraim et al., 2005). Such painful sensations are referred to as phantom limb pain (PLP) (Jensen et al., 1985). PLP includes a variety of different qualities such as burning, stabbing, or cramping (Katz and Melzack, 1990; Sherman, 1997; Giummarra et al., 2007). PLP must be differentiated from stump pain, which is characterized by painful sensations solely located in the residual limb. Furthermore, PLP also needs to be differentiated from non-painful sensations (phantom limb sensations) and the feeling of the enduring presence of the missing body part (phantom limb awareness) (Flor et al., 2006).

Previous studies have described structural and functional plasticity in different parts of the neuraxis following amputation (Flor et al., 2006). Functional plasticity was described in animal studies (Merzenich et al., 1984; Pons et al., 1991) as well as in humans (Weiss et al., 1998, 2000; Lotze et al., 2001; Flor et al., 2006) especially in primary somatosensory cortex (SI). It was assumed that sensory areas that formerly represented the amputated structure of the body became overtaken by the neural representations of neighboring body structures in SI. Flor et al. (1995) reported that the amount of such functional cortical reorganization is highly correlated with PLP intensity.

Beside this well replicated observation of functional cortical plasticity, additional neural plasticity of cortical volume was identified following amputation by Draganski et al. (2006) using voxel-based morphometry as well as by our research group (Preissler et al., 2013) using freesurfer. Thereby, amputation in upper limb amputees is associated with anatomical alterations in parts of the cortex that belong to dorsal and ventral visual streams (Preissler et al., 2013). Such plasticity was hypothesized to reflect a use-dependent increase of visual control in operating a hand prosthesis due to the fact that somatosensory information is usually not provided by most functional prostheses. Therefore, most amputees have to compensate this loss of information by increasing visual control of the prosthesis. A former national survey of 1,575 amputees addressed the lack of sensory feedback and the increased requirement of visual control by requesting “better control mechanisms that require less visual attention” (Atkins et al., 1996). Given that the technology has not changed dramatically since then, it still represents an important issue for most amputees with prostheses (for review see: Biddiss and Chau, 2007).

Cortical plasticity in the visual system might be a significant consequence of intensive visuomotor training to improve hand-eye coordination, as it was recently shown by Draganski et al. (2004). These authors used voxel-based morphometry to compare a group of volunteers before and after extensive juggling training across a period of 3 months. Before training, there was no structural difference within the visual system between the training group and a control group that received no juggling training. However, after 3 months, the juggling group showed selective structural increase of cortical volume in areas of the visual system. Transferring these observations to the situation of an arm amputee and prosthesis user, the increased visual control of the prosthetic device might also result in an increase of gray matter volume in the visual pathways of such individuals.

In line with this assumption is that we recently observed a gain in volume in the visual stream of arm amputees (Preissler et al., 2013) that might be a consequence of prosthesis use. There is empirical evidence that functionality of a prosthesis and amount of prosthesis use are negatively associated with PLP (Lotze et al., 1999; Weiss et al., 1999; Raichle et al., 2008; Dietrich et al., 2012). Interestingly, amputees with strong PLP showed less volume gain in the areas belonging to the visual stream (Preissler et al., 2013).

Therefore, it can be hypothesized that the extent to which vision is needed to provide feedback about the artificial hand depends on the quality and the quantity of prosthesis use and might be result in (feedback-dependent) cortical plasticity in the visual system. The aim of the study was to investigate the relationship between prosthesis use, PLP, and anatomical alterations in the visual system. More precisely, we hypothesize, first, a negative association between PLP and prosthesis use, at least in a sample of patients with strong PLP, and second, a relationship between the amount of prosthesis use and cortical volume in the visual stream of patients after upper limb amputation.

## Materials and Methods

### Participants

These data present a reanalysis of a published dataset (Preissler et al., 2013).

A sample of 21 patients amputated at their right upper limb participated in the study (age: mean: 44.5 years; min: 20 years, max: 62 years). Except for three female patients, all subjects were male. Exclusion criteria were plexus avulsion, amputations of further body parts, congenital malformation, and/or any neurological or psychiatric disorder (for sociodemographic and clinical data please refer to Table 1). Amputation was undertaken following trauma in 19 patients and due to sarcoma in two patients. The study was approved by the ethics committee of the Friedrich Schiller University. Informed consent was obtained from each subject prior to examination and participation in the study.

**Table 1:** **Demographic and clinical details of all 21 amputated patients**.

	Gender	Age in years	Side of amputation	Time since amputation in month	Stump length in cm	Cause of amputation	PLP rating (VAS)	BDI	Prosthetic use index	MPI_LI
1	M	32	r	19	ca. 45	Trauma	2.76	14	2	–
2	M	28	r	2	47	Trauma	0.50	2	16	1
3	M	43	r	180	63	Trauma	4.20	5	16	3
4	M	62	r	600	–	Sarcoma	1.40	8	8	3
5	M	24	r	29	37.5	Trauma	0.00	2	8	0
6	M	38	r	53	52.5	Trauma	2.45	30	16	2
7	F	56	r	133	–	Embolism	4.40	20	16	5
8	M	52	r	152	47	Trauma	5.20	8	1	5
9	M	27	r	14	42.5	Trauma	2.00	6	0	1
10	M	46	r	104	12	Trauma	9.00	12	12	5
11	M	20	r	1	11	Trauma	0.00	4	0	0
12	M	61	r	254	28	Trauma	0.00	1	6	2
13	M	53	r	230	14	Trauma	4.50	4	16	3
14	M	57	r	346	51	Trauma	0.00	6	16	1
15	M	59	r	59	26.5	Trauma	5.40	36	8	5
16	M	34	r	118	20	Trauma	0.00	5	16	2
17	M	59	r	11	46.5	Trauma	3.00	5	16	3
18	F	22	r	1	8	Trauma	6.05	40	0	–
19	M	57	r	60	71	Trauma	5.20	2	12	4
20	M	53	r	396	52	Trauma	7.50	17	16	6
21	F	51	r	105	40	Trauma	5.60	17	8	4

*PLP, phantom limb pain; VAS, visual analog scale; BDI, Becks Depression Inventory-II; MPI_LI, life interference scale of the Multidimensional Pain Inventory*.

### Assessment of phantom limb pain

The amputees were requested to rate the intensity of their PLP using a 10 cm Visual Analog Scale (VAS) with “no pain at all” represented by the left end point and “the strongest pain I can imagine” represented by the right end point of the scale (Scott and Huskisson, 1976).

### Assessment of prosthesis use

Participants were asked to rate their amount of prosthesis use on two scales. To describe their weekly amount of prosthesis use, the item “How often do you wear your prosthetic device within the week?” was utilized. For the rating a five point ordinal scale was used (0 = not at all, 1 = less than twice, 2 = every second day, 3 = nearly daily, 4 = every day). The second question focused on the daily amount of use (“How often do you wear your prosthetic device at each day?”). For this item, a five point categorical scale was used (0 = never, 1 = 1–2 h, 2 = several hours but not continuously, 3 = continuously the whole morning or afternoon, 4 = from morning to night). Since the prosthetic device might not be used every day, the overall amount of prosthesis use was expressed as the product of the subjects’ score for the weekly rating multiplied by the score of the daily rating.

### Assessment of life interference

In addition, subjects filled-in “The life interference scale” of the German version of the Multidimensional Pain Inventory (MPI; Kerns et al., 1985; Flor et al., 1990). The MPI is a questionnaire for multidimensional assessment of chronic pain. It consists of 52 items, which are subdivided into three sections (Kerns et al., 1985). The first section includes five scales that measure the patient’s perception of pain severity, life interference caused by chronic pain, experienced life control, affective distress, as well as social support. The participants have to respond on a 7-point scale ranging from 0 to 6.

### Assessment of depression symptoms

The subjects’ depression symptoms were assessed with the Beck Depression Inventory-II (BDI-II; Beck et al., 1996). The BDI-II is a 21-item questionnaire that assesses somatic, affective, and cognitive symptoms due to depression. Participants answer questions based on a 4-point scale ranging from 0 (not at all) to 3 (extreme form of each symptom) for the last 2 weeks. Scores range from 0 to 63 whereby scores of 30 and higher point to a “severe depression.”

### Group assignment

The participants were subdivided into a group with low to medium PLP (age: mean: 38.3 years, min: 20 years, max: 62 years) and into a second group with strong PLP (age: mean: 50.1 years, min: 22 years, max: 59 years). Group assignment was based on the impact PLP has on the patients’ daily life as measured by the life interference scale of the MPI according to a suggestion of Jensen et al. (2001).

Four ANOVAS with life interference as independent variable and mild versus severe pain as grouping variable were performed. The grouping variable was based on the VAS score (a) less than 2, (b) less than 3, (c) less than 4, (d) less than 5. Each model reached significance, with the second model with border VAS = 3 reaching the highest *F*-score [*F*(19, 1) = 24.86]. Based on these results, we divided our group into two subgroups, one with no to low PLP (VAS < 3) and the other with moderate to strong PLP (VAS ≥ 3).

Both groups do not significantly differ in the presented clinical and demographic variables besides as expected in PLP rating [low to medium PLP: *M* = 0.9; SD = 1.1; strong PLP: *M* = 5.5; SD = 1.6; *t*(19) = 7.32, *p* < 0.000] and experienced life interference [low to medium PLP: *M* = 1.5; SD = 1.1; strong PLP: *M* = 4.3; SD = 1.1; *t*(19) = 5.84, *p* < 0.000]. The groups do not differ in the amount of prosthetic use [low to medium PLP: *M* = 8.8; SD = 6.8; strong PLP: *M* = 11.0; SD = 6.1; *t*(19) = 0.44, *p* = 0.78].

### MRI data acquisition

For morphometric analyses, two T1-weighted sagittally oriented sequences were acquired for all subjects (192 slices; flip angle: 30°; matrix: 256 × 256; voxel size: 1 × 1 × 1 mm). As the participants began to be studied in 2005, the first eight subjects were measured on a 1.5-T MRI scanner (Siemens Magnetom Vision Plus, Erlangen, Germany; TE: 5 ms; TR: 15 ms) using a single-channel circulary-polarized (CP) head coil. For the remaining 13 participants, a 3-T MRI scanner (Siemens Magnetom Trio, Erlangen, Germany; TE: 3.03 ms; TR: 2.3 ms) with a 12-channel head matrix coil was used. Head movement was minimized using a vacuum pad.

### Cortical reconstruction and volumetric segmentation

Cortical reconstruction and volumetric segmentation was performed with the Freesurfer image analysis suite, which is documented and freely available for download online (http://surfer.nmr.mgh.harvard.edu/). Briefly, the processing includes motion correction and averaging (Reuter et al., 2010) of the two volumetric T1-weighted images, removal of non-brain tissue using a hybrid watershed/surface deformation procedure (Segonne et al., 2004), automated Talairach transformation, segmentation of the subcortical white matter and deep gray matter volumetric structures (Fischl et al., 2002, 2004), intensity normalization (Sled et al., 1998), tessellation of the gray matter-white matter boundary, automated topology correction (Fischl et al., 2001; Segonne et al., 2007), and surface deformation. The last procedure is accomplished by following intensity gradients to optimally place the gray/white and gray/cerebrospinal fluid borders at the location where the greatest shift in intensity defines the transition to the other tissue class (Dale and Sereno, 1993; Dale et al., 1999; Fischl and Dale, 2000). Once the cortical models are complete, a number of deformable procedures can be performed for further data processing and analyses including surface inflation (Fischl et al., 1999a), registration to a spherical atlas which utilizes individual cortical folding patterns to match cortical geometry across subjects (Fischl et al., 1999b), parcellation of the cerebral cortex into units based on gyral and sulcal structure (Fischl et al., 2004; Desikan et al., 2006), and creation of a variety of surface based data including maps of curvature and sulcal depth. This method uses both intensity and continuity information from the entire three-dimensional MR volume in segmentation and deformation procedures to generate representations of cortical thickness, calculated as the closest distance from the gray/white boundary to the gray/cerebrospinal fluid boundary at each vertex on the tessellated surface. Freesurfer morphometric procedures have been demonstrated to show good test-retest reliability across scanner manufacturers and across field strengths (Han et al., 2006). Volume measures may be mapped on the inflated surface of each participant’s reconstructed brain. Maps are smoothed using a circularly symmetric Gaussian kernel across the surface with a standard deviation of 10 mm and averaged across participants using a non-rigid high-dimensional spherical averaging method to align cortical folding patterns. This procedure provides accurate matching of morphologically homologous cortical locations among participants, resulting in a mean measure of cortical thickness for each group at each point on the reconstructed surface. The entire cortex in each participant was visually inspected and any inaccuracies in segmentation were manually corrected by experts in brain anatomy who were blind to group membership.

Statistical comparisons of global data and surface maps were generated by computing a general linear model of the effects of amount of prosthesis use on volume at each vertex. Cortical volume clusters were first displayed using a threshold that shows all vertices with *p-*values below 0.005. Additionally, only clusters with a minimum size of 10 vertices were accepted (Lieberman and Cunningham, 2009).

### Statistics

Apart from the Freesurfer analysis suite, statistical analyses were performed using IBM SPSS Statistics 20 (version 20.0, SPSS Inc., an IBM Company, Chicago, IL, USA). As the measure of prosthesis use is not a continuous variable, correlations were determined using a non-parametric measure of statistical dependence (Kendall’s tau; τ).

## Results

### Behavioral analysis

We found no relation between PLP and the amount of prosthesis use for the whole patient group (τ = −0.023; *p* = 0.448). There was no relation between the aforementioned variables in the group with low PLP (τ = −0.111; *p* = 0.345); however, in the group with strong PLP, PLP was significantly associated with less prosthesis use (τ = −0.415; *p* = 0.049).

### Morphometric analyses

#### All patients

**Figure 1:**
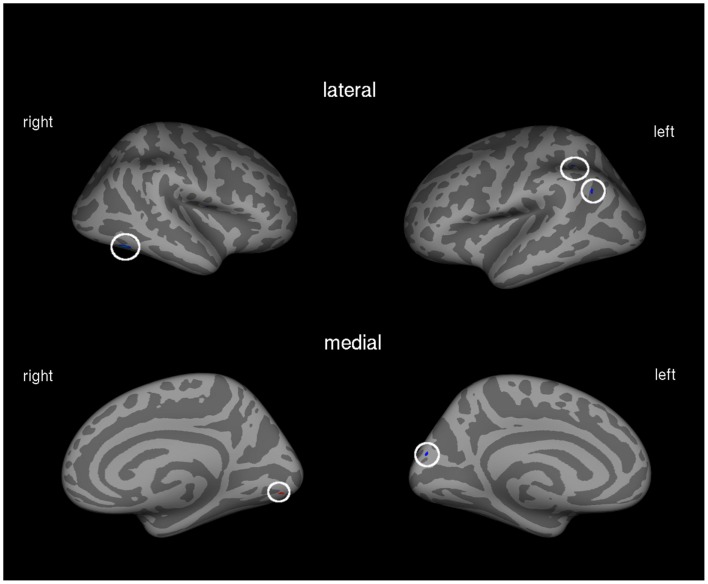
**Correlation between amount of prosthesis use and cortical volume in the total patient group (*N* = 21)**. Blue areas indicate negative associations, areas in red show positive associations.

We found correlations between the amount of prosthesis use and gray matter volume in different cortical areas, especially in parts of the left posterior parietal lobe, as well as a positive association between cortical volume of the right visual association cortex and the amount of prosthesis use (see Table 2; Figure 1).

**Table 2:** **Areas with correlational relation between the reported amount of prosthesis use and cortical volume for all patients (*N* = 21)**.

Region	Subregion		Talairach coordinates			Cluster size	DCA	*Z*-score
	
			X	Y	Z	
Parietal lobe	LH	Intraparietal sulcus	−37.7	−46.1	34.5	35	−	3.24
		Superior parietal sulcus	−43.2	−58.2	27.5	29	−	3.40
Temporal lobe	RH	Middle temporal gyrus	42.4	−57.8	−9.6	86	−	4.20
Occipital lobe	LH	Cuneus, BA 18	−3.6	−79.6	21.2	20	−	3.24
	RH	Lingual gyrus, BA 18	10.5	−85.7	−12.7	21	+	3.22

*LH, left hemisphere; RH, right hemisphere; BA, Brodmann area; DCA, direction of cortical association; −, negative correlation between cortical volume and amount of prosthesis use; +, positive association between cortical volume and amount of prosthesis use*.

#### Patients with low to medium phantom limb pain

**Figure 2:**
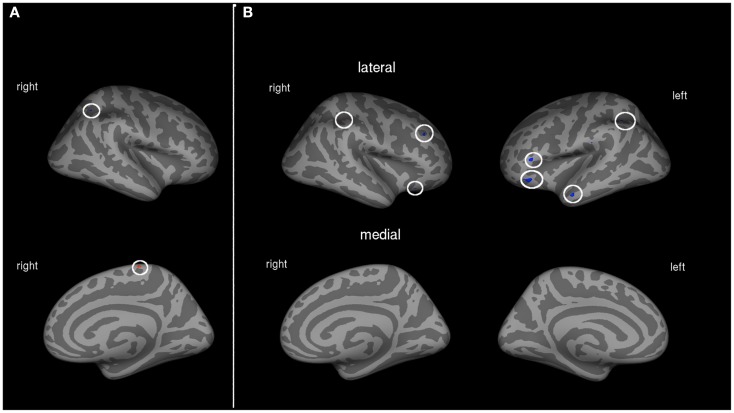
**(A)** Correlation between amount of prosthesis use and cortical volume in patients with low to medium PLP (*N* = 10). Blue areas indicate negative associations, areas in red show positive associations. **(B)** Correlation between amount of prosthesis use and cortical volume in patients with strong PLP (*N* = 11). Blue areas indicate negative associations, areas in red show positive associations.

Patients with low to medium PLP showed associations between cortical volume and amount of prosthesis use in two areas of the cortex. A region of the right posterior parietal lobe was negatively, and a part of the paracentral sulcus was positively associated with the amount of prosthesis use (see Table 3; Figure 2A).

**Table 3:** **Areas with correlational relation between the amount of prosthesis use and cortical volume for patients with low to medium phantom limb pain (*N* = 10) and for patients with strong phantom limb pain (*N* = 11)**.

Subregion			Talairach coordinates			Cluster size	DCA	*Z*-score
	
			X	Y	Z	
**PATIENTS WITH LOW TO MEDIUM PHANTOM LIMB PAIN**								
Parietal lobe	RH	Intraparietal sulcus	31.6	−55.2	40.3	20	−	3.47
Frontal lobe	RH	Paracentral	3.7	−29.0	66.9	34	−	3.93
**PATIENTS WITH STRONG PHANTOM LIMB PAIN**								
Parietal lobe	LH	Intraparietal sulcus (posterior)	−38.7	−49.2	34.3	26	−	3.19
	RH	Intraparietal sulcus	40.7	−40.8	35.5	15	+	3.59
Temporal lobe	LH	Middle temporal gyrus	−55.9	−3.4	−25.9	45	−	3.73
Frontal lobe	LH	Inferior frontal gyrus	−43.1	28.6	−14.2	113	−	3.59
		Inferior frontal gyrus	−52.3	29.3	6.0	57	−	3.57
	RH	Inferior frontal gyrus	30.7	23.4	−20.1	11	−	3.17
		Rostral middle frontal gyrus	36.6	28.7	31.8	29	−	3.43

*LH, left hemisphere; RH, right hemisphere; DCA, direction of cortical association; −, negative correlation between cortical volume and amount of prosthesis use; +, positive association between cortical volume and amount of prosthesis use*.

#### Patients with strong phantom limb pain

Cortical volume of patients with strong PLP was negatively associated with the amount of prosthesis use in the left posterior parietal lobe, left middle temporal gyrus, and parts of the dorsolateral prefrontal cortex (DLPFC) on both hemispheres. The volume of a single cortex region showed a positive association with the amount of prosthesis use: the right intraparietal sulcus (see Table 3; Figure 2B).

#### Influence of covariates

To ensure that the results mentioned above are not caused by the different field power of the two different MRI scanners or age of the participants, we performed additional partial correlations with scanner type, or age as covariate, respectively. None of these covariates had a significant influence on the results.

## Discussion

The aim of this study was to investigate the relationship between prosthesis use, PLP, and cortical volume plasticity in the visual system. On the behavioral level, a significant correlation between PLP and prosthesis use was found in patients with strong PLP. Only in these patients stronger PLP was associated with less use of the prosthesis. This association was neither significant for the whole group of patients nor for the subgroup with low PLP.

Correlation analyses revealed significant associations between the amount of prosthesis use and the cortical volume of brain regions that are part of the visual streams for all patients as well as in the patient group with strong PLP.

### Behavioral analysis

Previous studies have already shown that the amount of prosthesis use is associated with PLP reduction. Thus, Weiss et al. (1999) investigated nine patients using a Sauerbruch prosthesis and 12 persons wearing cosmetic prostheses. The authors demonstrated that the use of the “active” Sauerbruch prosthesis is accompanied by a reduction of PLP, whereas the use of the cosmetic prosthesis had no impact on PLP; four of the 12 patients even reported stronger PLP. These results indicate that patients who use their residual limb with the prosthesis more often in daily life show less PLP. This finding confirms results of a recent study of our research group showing that “active” functional hand prostheses with additional somatosensory feedback lead to further decrease in PLP (Dietrich et al., 2012).

Our results are also in accordance with a study by Lotze et al. (1999). These authors showed that the relationship between PLP and functional cortical reorganization in SI was mediated by prosthesis use. Patients who were not provided with a functional myoelectric prosthesis showed stronger PLP and a significant larger extent of functional cortical reorganization than patients using a myoelectric prosthesis. Lotze et al. (1999) suggested that beside ongoing stimulation by the artificial device and muscular training, visual feedback from the artificial device might have a beneficial effect on functional cortical plasticity as well as on PLP. This goes in line with our former publication in which persons with strong PLP showed less cortical volume in areas belonging to the visual stream (Preissler et al., 2013).

Moreover, there is an effective therapy approach focusing on visual feedback, the mirror visual feedback therapy (Ramachandran et al., 1996; Ramachandran and Altschuler, 2009). In this therapy, a mirror is placed next to the patient’s residual limb. Thereby, the mirror image creates an illusion of an intact limb (Ramachandran and Rogers-Ramachandran, 1996). The patients reported that they felt their amputated limb return. In most patients this vivid perception of their former limb seems to be followed by a reduction in PLP (Ramachandran and Rogers-Ramachandran, 1996; Ramachandran and Altschuler, 2009). An alternative approach which demonstrated that visual input is able to change somatic sensation is the rubber hand illusion (Botvinick and Cohen, 1998; Bekrater-Bodmann et al., 2012). Like mirror therapy it uses the visual but illusory replacement of the missing hand. To produce this illusion, a rubber hand is placed in front of the participant in a position that equals the perceived phantom hand of the amputee. A third therapeutic approach focusing partly on visual feedback is graded motor imagery (Moseley, 2004, 2006; Johnson et al., 2012). This training was developed for patients with complex regional pain syndrome. It aims at reducing pain by recognition, imaging and moving of the affected limb (Moseley, 2004). In the first stage, patients view photographs of a left or right hand in a variety of postures and are asked to decide whether a left or right hand is shown. These approaches demonstrate that the integration of visual feedback by means of photographs (Moseley, 2006), a mirror (Ramachandran and Altschuler, 2009; Diers et al., 2010), or a rubber hand (Ramakonar et al., 2011; Bekrater-Bodmann et al., 2012) can reduce chronic pain and PLP. This indicates that visual feedback modulates somatic, especially painful sensations. Therefore, our behavioral data suggest that the more the artificial arm is used, the more the visual system might be engaged, and the less PLP is developed. Surprisingly this only seems to hold for the group with strong PLP.

Only in amputees with strong PLP the amount of prosthesis use was negatively associated with PLP. However, as the paradigm of the present study was designed cross-sectionally, it cannot be differentiated whether the increased PLP is a consequence of the reduced prosthesis use or whether PLP affects the patients’ behavior that, in turn, results in the reduced use of the artificial device. The relationship between PLP and the amount of prosthesis use might be non-linear.

### Morphometric analyses

#### All patients

Based on the behavioral results, we suppose that structural cortical plasticity is associated with the use of the functional hand prosthesis. Such structural plasticity has been observed in many cortical areas and, according to previous observations of our research group, might also happen in the visual system when the use of the prosthesis requires increased visual control of its operation.

We found associations between cortical volumes and the amount of prosthesis use in several cortical areas. Negative associations were found in parts of the left posterior parietal cortex (intraparietal sulcus, and superior parietal sulcus), in the cuneus, and in the right middle temporal gyrus. Only the right lingual gyrus, as part of the visual association cortex, showed a positive association between volume and prosthesis use.

Visual areas in the ventral and dorsal pathways are critical areas for pattern recognition and the segmentation, classification, localization, and handling of the visual objects (Roelfsema, 2006; Kravitz et al., 2011). The dorsal stream includes occipital, posterior-temporal, and parietal regions and is mainly involved in the processing of spatial orientation of objects, the detection of movements, and eye-hand coordination. The ventral stream is located in occipital and temporal lobes; it is mainly involved in object perception (Roelfsema, 2006; Cavina-Pratesi et al., 2010; Kravitz et al., 2011; Monaco et al., 2011). The associations between the amount of prosthesis use and cortical volume are located in areas belonging to the dorsal stream.

One of the most striking results of the present investigation following upper limb amputation is the negative association between prosthesis use and cortical volume in posterior parts of the parietal lobe (intraparietal sulcus, superior parietal sulcus). We observed increasingly smaller volumes the more the amputees wore and used their prosthetic device. As part of the dorsal stream, these brain areas are important for hand orientation and eye-hand coordination in grasping (Castiello and Begliomini, 2008; Monaco et al., 2011). Since fine tuning of the prosthesis during grasping is lost due to the fact that the prosthetic devices used by the patients of our study did not include a flexible wrist, all patients lost the functional control of the wrist. This loss of wrist coordination might have been followed by a reduction of cortical volume in regions which formerly coordinated wrist movement.

The cortical volume of the intraparietal sulcus of the parietal lobe was negatively correlated to the amount of myoelectric prosthesis use. This area contains cells that organize convergence of vision and proprioception and enables subjects to perceive where objects are located in the peripersonal space (Hyvarinen and Shelepin, 1979; Hyvarinen, 1981). However, besides visual feedback, a myoelectric hand prosthesis does not provide somatosensory feedback. The more often the patients use their functional hand prosthesis, the more frequently they learn that proprioceptive and visual information do not converge during grasping which might be accompanied by a reduction of volume in the intraparietal sulcus.

The patients also showed a negative association between the amount of prosthesis use and the volume of the left cuneus. The cuneus is part of the visual association cortex. It is known to be involved in bottom-up, stimulus-driven control of visuo-spatial selective attention, e.g., it is engaged by stimulus-driven orienting (Hahn et al., 2006). However, prosthesis use is much more dominated by top-down control since patients need to plan ahead their movements intentionally (Atkins et al., 1996). It can be reasoned that with increasing prosthesis use, patients rely more often on top-down processes instead of bottom-up control. Amputees may be forced to focus their attention to their prosthetic device while using it, and therefore might pay less attention to their surroundings, and, as a result, show less cuneus activation. This may cause a diminished engagement of the cuneus. Therefore, the negative correlation between prosthesis use and the volume of the cuneus might represent a reduced functional use of this area accompanied by elevated use of the prosthetic device.

In addition to the described negative associations between cortical volume and prosthesis use, the right lingual gyrus showed a positive relationship with the amount of prosthesis use. For this part of the visual association cortex, Schiltz et al. (1999) showed an orientation-specific function of the lingual gyrus. They studied the effect of extensive training in a visual orientation discrimination task. Orientation-specific information is very important for individuals who need to use an artificial device during grasping. Unlike healthy people, they cannot change the position of the hand while grasping spontaneously. Therefore, persons with upper limb amputation need to plan the grip process further ahead. Thus, orientation-specific information of an object’s position gains more importance. The amputee needs to focus on the object as well as on how to approach it before he/she starts grasping the object. More extensive use of the artificial device requires a more detailed processing of the object-specific orientation information. This can be followed by a use-dependent increase in cortical volume of areas that process such orientation-specific information. Hence, the patients show more volume of the right lingual gyrus, the more they actively used their artificial device.

#### Patients with strong phantom limb pain

Patients with strong PLP showed significant associations between prosthesis use and cortical volumes in a larger amount of areas than patients with low PLP. These associations indicate that PLP is accompanied by more cortical alterations than the amputation itself which is in line with previous data on anatomical alterations (Makin et al., 2013; Preissler et al., 2013). In patients with strong PLP, the amount of prosthesis use was negatively correlated with the cortical volume of different areas corresponding to the dorsal and ventral visual streams. Surprisingly, these alterations were negative, i.e., patients with strong PLP showed more cortical volume when prosthesis use was less frequent. Since we hypothesized that less frequent use of the prosthesis will result in lower necessity of visual control of the artificial hand, these findings seem to be contra-intuitive. However, it might well be that the brain is forced to adapt to the new requirements entailed by prosthesis use as explained in the previous paragraphs. Moreover, on the behavioral level we found that less prosthesis use was associated with stronger PLP within this group of strong PLP patients. As the PLP level in this group was constantly stronger, it might have an indirect influence on the cortical volume of the visual streams.

The most striking part of the visual stream which is negatively correlated to the frequency of prosthesis use is located in the left posterior parietal cortex, the intraparietal sulcus. This might reflect an adaptation to the use of an artificial device which substitutes a few functions of the former hand (e.g., wrist orientation, which is discussed extensively in the former section of discussion). The more often a prosthesis is worn, the more the brain adapts to the new movement patterns as it gains more experience with regard to functions of the former hand which are not necessary anymore to guide the artificial hand, and others which are still useful. Therefore, this might be an area showing a use-dependent specific adaptation. Interestingly, this association mirrors the result in the group of all patients, but was not existent in patients with low PLP. Taken into account that higher prosthesis use is associated with lower PLP in patient group with strong PLP, this might indicate an adaptation process existing in patients with strong PLP but not in patients with low PLP.

#### Patients with low to medium phantom limb pain

In patients with low to medium PLP, only two of the analyzed brain areas showed associations with the amount of prosthesis use. One area that is part of the right intraparietal sulcus was negatively correlated with the amount of prosthesis use. The second region where associations between the amount of prosthesis use and cortical volume could be shown is the paracentral sulcus. The cortical volume in this area was positively associated with the amount of prosthesis use.

The negative association between the cortical volume in the right intraparietal sulcus as part of the posterior parietal cortex, and prosthesis use contrasts the result for the group with strong PLP. It supports the hypothesis that the prosthesis use differs qualitatively as a function of PLP and might indicate that patients with lower PLP need another amount of visual attention in their prosthesis use than patients with strong PLP. This is potentially reflected by the different associations between cortical volume and amount of prosthesis use in the right intraparietal sulcus.

There is a part of the paracentral sulcus where patients with low PLP showed more cortical volume when they used their prosthesis more frequently. This region is known as the supplementary eye field (Grosbras et al., 1999). It is located at the most rostral part of the supplementary motor area. This region is important for the control of eye movements. The association between the cortical volume and the prosthesis use was found right-sided. This goes in line with an assumption of Grosbras et al. (1999) that the right supplementary eye field is required for aspects of visual guidance. Using a prosthesis requires permanent visual guidance (Atkins et al., 1996; Biddiss and Chau, 2007). This correlation between cortical volume of the right paracentral sulcus and prosthesis use might reflect the greater demand of visual control in patients who need upper limb prostheses.

### Limitations and future directions

Our study has several limitations. The sample size is small. As we studied a rare patient group and due to technical development in the course of this research project the patients were measured on two different MRI scanners with different field strengths, this might, even if controlled for, induce a bias. However, none of the patients was scanned on two different scanners longitudinally, so that all changes are related to differences on the same scanner. Nevertheless, results should be interpreted with caution.

The quality of prosthesis use should be assessed in future studies as well as the quantity of prosthesis use to account for potential influences of both. Furthermore, we can only make limited conclusions regarding the dynamics of morphological brain changes over time. Further research with a longitudinal design might help to account more properly for changes over time on the behavioral level as well as for morphometric changes. Such studies would probably allow to access use-dependent cortical plasticity which may have vast clinical implications for the treatment of chronic pain.

## Conclusion

Our behavioral data support the hypothesis that PLP might change as a function of increased prosthesis use. This association is strongly expressed in the group of patients suffering from strong PLP. Unfortunately, we cannot reason whether prosthesis use is followed by less PLP or strong PLP leads to a decrease of prosthesis use.

Based on the amputees’ behavior, we differentiated structural cortical alterations connected to the amount of prosthesis use *per se* and those that reflect the intensity of PLP. Structures that showed volumetric plasticity are regions associated with both, the requirements of the use of a functional myoelectric hand prosthesis and visual control.

The relation between amount of prosthesis use and cortical volume in the left posterior parietal cortex as part of the dorsal stream seems to be specific for the group of patients with strong PLP. Given that we could give evidence that prosthesis use seems to be differently associated to changes in the visual streams in patients with low PLP compared to patients with strong PLP.

## Conflict of Interest Statement

The authors declare that the research was conducted in the absence of any commercial or financial relationships that could be construed as a potential conflict of interest.
